# Assessment of toxicity and anti-plasmodial activities of chloroform fractions of *Carapa procera* and *Alchornea cordifolia* in murine models

**DOI:** 10.3389/fphar.2022.1077380

**Published:** 2022-12-23

**Authors:** Ayisha Mahama, Mary Anti Chama, Emelia Oppong Bekoe, George Awuku Asare, Richard Obeng-Kyeremeh, Daniel Amoah, Constance Agbemelo-Tsomafo, Linda Eva Amoah, Isaac Joe Erskine, Kwadwo Asamoah Kusi, Samuel Adjei

**Affiliations:** ^1^ West Africa Centre for Cell Biology of Infectious Pathogens, University of Ghana, Accra, Ghana; ^2^ Department of Chemistry, School of Physical and Mathematical Sciences, College of Basic and Applied Sciences, University of Ghana, Accra, Ghana; ^3^ Department of Pharmacognosy and Herbal Medicine, School of Pharmacy, College of Health Sciences, University of Ghana, Accra, Ghana; ^4^ Department of Medical Laboratory Sciences, School of Biomedical and Allied Health Sciences, College of Health Sciences, University of Ghana, Accra, Ghana; ^5^ Department of Animal Experimentation, Noguchi Memorial Institute for Medical Research, College of Health Sciences, University of Ghana, Accra, Ghana; ^6^ Department of Immunology, Noguchi Memorial Institute for Medical Research, College of Health Sciences, University of Ghana, Accra, Ghana; ^7^ Department of Pathology, Korle Bu Teaching Hospital, Accra, Ghana

**Keywords:** *Alchornea cordifolia*, *Carapa procera*, chloroform fraction, anti-plasmodial, toxicology

## Abstract

**Background:** Plant as a source of medicine has gained international popularity in recent times because of its natural origin, availability in local communities, cheaper to purchase, ease of administration, and its usefulness as an alternative treatment in case of numerous side effects and drug resistance. However, the use of herbal formulations can also result in short-term and long-term organ damage or dysfunction to the host. In this study, chloroform fractions of the leaves of two medicinal plants, *Alchornea cordifolia* (ACL) and *Carapa procera* (CPL), were investigated for their toxicological and anti-malarial effects in murine models.

**Method:** Acute (14-day) and sub-acute (28-day) studies were conducted based on the Organization for Economic Cooperation and Development (OECD) Guidelines in Institute for Cancer Research (ICR) mice and Sprague Dawley (SD) rats respectively. A dosage of 2000 mg/kg body weight was administered orally to each ICR mouse during the acute study and 100, 300, and 1000 mg/kg body weight to each SD rat during the sub-acute study. A 5-day curative anti-plasmodial activity was assessed in ICR mouse model.

**Results:** The assessment of toxicity revealed that all three fractions did not influence mortality, clinical appearance, body weight gain, or necropsy at the various doses. Hematological and serum biochemical analysis indicated no significant elevations in liver and renal function parameters. Histopathological examinations of the liver indicated reversible liver degeneration with the chloroform fraction of the 100% ethanol extract of *Carapa procera* leaves (CPL100%) at 1000 mg/kg. Anti-plasmodial assessments showed CPL100% exhibiting dose-dependent anti-plasmodial activity from 16% to 26.67%. On the other hand, chloroform fraction of the 100% ethanol extract of *Alchornea cordifolia* leaves (ACL100%) showed declining anti-plasmodial activity from 21.1% to 15.1%.

**Conclusion:** These preliminary findings demonstrate that chloroform fractions of the leaves of *Carapa procera* and *Alchornea cordifolia* may be safe agents for treating malaria hence further development for drug discovery must be pursued.

## 1 Introduction

Antimalarial drug resistance to chloroquine and now artemisinin derivatives is the impetus that drives the search into novel antimalarials (Miller et al., 2013). The search for new antimalarials has led scientists into investigating microbes, animal fur and plant extracts for antimalarial activity (Martínez-Luis et al., 2012; Durant et al., 2014; Higginbotham et al., 2014; Weber et al., 2019). In poor and developing countries, healthcare is frequently supported by indigenous medicinal herbs, with up to one-fifth of patients using indigenous medicines for the treatment of diseases including malaria (Willcox and Bodeker, 2005). Around 80% of African rural people rely on traditional herbal treatments (Li et al., 2005). However, adverse health effects have been associated with the usage of some herbal medicines. These effects range from cardiovascular, neurological, hepatic, and renal toxicity problems (Ernst, 2003a; Ernst, 2003b; Tovar and Petzel, 2009).

*Carapa procera*, DC. (Meliaceae) is a renowned antimalarial used in Africa, India and tropical America (Schwikkard and van Heerden, 2002) mostly for gametocidal and antiplasmodial activities (Santos et al., 2022). All parts of the plant (root, bark, nuts, mistletoe and, leaves) are exploited for medicinal and cosmetic purposes (Tuchscherer et al., 2012). The leaves and stem bark of C. procera are popular herbs used by traditional healers in Burkina Faso and across West Africa for the treatment of malaria (Koama et al., 2021).

*Alchornea cordifolia*, (Schumach and Thorn) Mull. Arg. belonging to the family Euphorbiaceae is locally known as Ogyama *locally known in Ghana as Ogyama and* is used to treat malaria and other disease such as rheumatic pain, stomach ulcers and parasitic infections (Agbor et al., 2004; Mavar-Manga et al., 2008; Adeshina et al., 2009; Soh et al., 2009; World Health Organization, 2018). The plant which is a shrub has its leaves boiled alone or together with other plants to form a decoction, that is, drank three times daily for the treatment of malaria (Asase and Asafo-Agyei, 2011). The young shoots of Alchornea cordifolia are reported to be used to treat malaria by Baka pygmies of the East and South provinces of Cameroon (Zofou et al., 2013).

This study investigated the toxicological and antimalarial effects of the chloroform fractions of the leaves of *Carapa procera* and *Alchornea cordifolia* in murine animal models*.* The chloroform fraction, though not related to the traditional use, the investigators deemed it necessary to investigate various fractions of varying polarity to enable the determination of the most active as part of efforts to validate the usefulness of these plants as antimalarials. Acute and sub-acute toxicological studies were performed in murine models, to identify adverse toxic effects of the fractions followed by investigations into whether the fractions from these plants cause *in-vivo* suppressive anti-malarial effects in mice infected with *Plasmodium berghei.*

## 2 Materials and methods

### 2.1 Fractionation of plant extracts

The chloroform fractions of the hydro-ethanol extracts of *Alchornea cordifolia* leaves (ACL1:1), absolute ethanol extracts of *Alchornea cordifolia* leaves (ACL100%) and absolute ethanol extracts of *Carapa procera* leaves (CPL100%), were chosen for this study based on unpublished data from a larger project which previously identified these fractions as having *in-vitro* anti-plasmodial activities. *Alchornea cordifolia* leaves (ACL) were collected from Shama Ahanta East of the Western region of Ghana (Latitude: 4.9074, Longitude: −1.8185) on December 6, 2018, and on February 18th, 2019, while *Carapa procera* leaves (CPL) were obtained from Abotakyi, near Mampong Akwapim of the Eastern region of Ghana (GPS Coordinates N 05^o^ 55.932s; W 00^o^ 07.914s) on December 6, 2018. They were identified by taxonomist Patrick Ekpe of the Department of Plant Science and Environmental Biology, University of Ghana. Voucher specimen of the plants, AC-001 and CP-001 have been prepared and deposited at the National Herbarium, University of Ghana. The plant materials were air-dried and milled*.* An amount of 250 g of each of the dried plant materials were soaked overnight in 100% ethanol as in the cases of the ACL100% and CPL100% fractions and 50% ethanol and 50% water to make a 1:1 concentration, as in the case of ACL1:1 fraction, and allowed to macerate for 24 h. Each extract was then decanted into a beaker and filtered through a glass funnel with cotton wool. The acquired ACL1:1, ACL100% and CPL100% extracts were further fractionated sequentially using separating funnels. The fractionations were done by the separation funnel method as described previously (Ingle et al., 2017). Fractionation began in petroleum ether, repeated about 5 times, until a clear petroleum ether layer was achieved. This was followed by transfer of the collected petroleum ether fractions into separating funnel and were fractionated in chloroform and repeated until extraction was complete. The aqueous layers of ACL1:1, ACL100% and CPL100% referred to as chloroform fractions were collected into beakers. These aqueous layers were then concentrated using a COLE-PARMER rotary evaporator, at 40°C and the recovered solvents were collected into labeled conical flasks. Recovered solvents were used for the next round of fractionation. Concentrated fractions were left to air dry, after which they were weighed to obtain a uniform mass.

## 3 Experimental animals

Healthy young adult animals were used in this study. A total of 65 female Institute of Cancer Research mice (ICR mice) aged 6–8 weeks and weighing 20–30 g, and 12 female Sprague Dawley rats (SD Rats), aged 6–8 weeks and weighing 120–150 g, were used in the study. The animals, bred and maintained in the Laboratory Animal Facility of the Department of Animal Experimentation, were kept in normal rodent cages with sterilized soft wood shavings as bedding. They were fed rodent feed pellets (AGRIFEEDS, Kumasi, Ghana), given clean water *ad libitum*, and kept under regulated laboratory conditions (Temperature 23°C ± 2°C, relative humidity 60%–70%, and a 12-h light-dark cycle).

### 3.1 Toxicological assessment of fractions

Toxicity assessment was conducted using female mice and rats as per the Organization for Economic Co-operation and Development (OECD) guidelines test numbers 420 (Organisation for Economic Co-operation and Development, 2002) and 407 (Organisation for Economic Co-operation and Development, 2008) for acute and sub-acute oral toxicity studies, respectively.

#### 3.1.1 Preparation of animals

All animals used in the study were uniquely identified and kept in their cages for seven (Li et al., 2005) days prior to the start of the study to allow for acclimatization to the laboratory conditions. Cages were labelled per respective groups arranged serially and well-spaced to minimize any cage placement effect. In the acute toxicity study, 20 female ICR mice were weight-matched into four groups (*n* = 5), three experimental groups and a control group. In the sub-acute toxicity study, the 12 female SD rats, were weight-matched and grouped into four cages (*n* = 3).

#### 3.1.2 Acute toxicity studies

A one-time dose of 2000 mg/kg body weight of each of the test fractions, ACL1:1, ACL100% and CPL100%, was administered orally to each mouse in the test group while 10% DMSO, which was used as the vehicle, was administered to the control group, post 3 h of fasting. The animals were observed immediately after administration of the extract, and closely monitored every 30 min for the first 4 h for behavior, signs of toxidromes and mortality. Subsequently, the animals were examined daily for clinical signs of toxidromes for 14 days while body weight was recorded weekly. Animals were euthanized (using isoflurane) on day 15, blood was collected *via* cardiac puncture for hematological and serum biochemical analyses. Livers and kidneys were harvested following gross necropsy for histopathological processing.

#### 3.1.3 SUB-ACUTE toxicity studies

A repeated 28-day oral toxicity was conducted. For each of the test fractions, doses of 100 mg/kg, 300 mg/kg and 1000 mg/kg were administered to the rats in test groups one, two and three, respectively, while the 4th group was given 10% DMSO, post fasting overnight. The rats were examined daily prior to the daily administration of the respective fraction’s dosage for 28 days. Rats were weighed weekly, and the dosages were adjusted accordingly. Animals were euthanized (using isoflurane) on day 29, blood was collected *via* cardiac puncture for hematological and serum biochemical analysis. Livers and kidneys were harvested following gross necropsy for histopathological processing.

#### 3.1.4 Gross necrospy examination

The animals were euthanized by placing them in a glass chamber with tissue soaked with isoflurane till cessation of breathing. The state of the fur, natural orifices’ excretions and secretion, condition of various body regions, including the head, neck, and chest as well as the state of the external genitalia and visible mucous membrane’s state was first assessed. Dorsal recumbency was used to position and pin the animal unto the dissection board. Reflected and fixed to the dissection board were the front and hind limbs. The animal’s abdomen was deskinned, and the abdominal cavity was opened just below the ribs. The diaphragm was checked for inrush of air (there should be negative pressure unless there has been pneumothorax or other significant pulmonary lesions). The rib cage was cut on the lateral ends to reveal the thoracic cavity. All organs were examined to evaluate their position *in-situ*. The mouth was then opened to check for lesions and the intermandibular space slit open caudally. The trachea with the esophagus was removed by cutting the soft tissue around the neck. With continued traction the entire pluck was removed by cutting connections in the thoracic cavity holding the lungs and heart in place. The esophagus and trachea were examined for froth in their lumen and any other abnormalities. The visceral organs including the lungs, heart, liver, kidneys, spleen were examined based on their size, consistency, color, and appearance of cut surface. The gastrointestinal tract, genital tract and bladder was also examined for abnormalities. Samples of the visceral organs were collected and stored in 10% buffered formalin.

#### 3.1.5 Haematological assessment

Blood samples, of about 0.5–1 ml, were collected into EDTA tubes and analyzed immediately, using the SYSMEX hematology autoanalyzer (Kobe, Japan). Leukocyte count, erythrocyte count, hemoglobin concentration, hematocrit, mean corpuscular volume (MCV), mean corpuscular hemoglobin (MCH), mean corpuscular hemoglobin concentration (MCHC), reticulocyte ratio, platelet count and differential leukocyte counts were determined.

#### 3.1.6 Serum biochemistry assessment

Blood samples of about 0.5–1 ml were collected into serum separator tubes and centrifuged at 6000 rpm for 15 min to separate the serum from plasma. Serum samples were then transferred into cryovials for immediate analysis. The following biochemical assays were performed using the SELECTRA JUNIOR Version 04 autoanalyzer (Vital Scientific, Spankeren, Netherlands): total bilirubin (TBIL), direct bilirubin (DBIL), aspartate aminotransferase (AST), alanine aminotransferase (ALT), total protein (TP), albumin (ALB), globulin (GB), alkaline phosphatase (ALP), ɣ-glutamyl transpeptidase (ɣ-GT), urea (URE), creatinine (CR).

#### 3.1.7 Histopathological examination

Following gross necropsy, the kidneys, liver, lung, spleen, brain, and heart were harvested. Wet organ weights were measured. Organs were then fixed with 10% buffered formalin. Formalin-fixed liver and kidney tissue samples were processed for histological examination as described previously (Khamaisi et al., 1997). Concisely, the samples were dehydrated in alcohol series (70%, 80%, 90%, and 100%) followed by further dehydration in xylene. Following the dehydration step, the samples were embedded in molten paraffin wax and then cut from a similar site of the tissue and in a similar plane at 4 μm thick. Next, samples were rehydrated in a decreasing series of alcohol and distilled water and then stained with haematoxylin & eosin (H&E) to evaluate morphological changes.

### 3.2 *IN VIVO* antiplasmodial assessment of fractions

#### 3.2.1 Inoculum preparation

Cryopreserved *Plasmodium berghei* at 5% parasitized red blood cells (prbcs) was thawed and intraperitonially injected with 200 μl into each donor mouse. The mice were monitored daily for the establishment of parasitemia. Parasitemia was then calculated by counting the number of prbcs against total red blood cells. Once parasitemia reached 5%, the donor mice, was euthanized and blood was collected *via* cardiac puncture, using a sterile syringe and into a heparinized tube. Dilution of the blood was done using 0.9% saline to produce 1 ml blood containing 1 × 10^7^ *P. berghei* infected RBC. 0.2 ml of the infected blood was subsequently administered intraperitoneally to each of the test mice.

#### 3.2.2 Curative test

The curative test, as described by Ryley and Peters (Ryley and Peters, 1995) was used to evaluate the curative capability of the extracts on established *P. berghei* infections. Female mice, known to be more susceptible to *Plasmodium berghei* infection than males, were used in the study. A donor group of five mice, randomly selected from the group of mice selected for the study, were assessed for parasitemia, and were found to be free of the malaria parasites. Assessment was done by preparing a thin smear of blood obtained by tail clipping. The thin blood smears were giemsa stained and observed under a compound light microscope at x100 magnification using oil immersion. Subsequently, each of the donor mouse was inoculated with 1 × 10^7^ *P. berghei* (fixed for all inoculations) and parasitemia was assessed 72 h post-inoculation and repeated daily until parasitemia was established. The *Plasmodium berghei* parasitized donor mice were then euthanized, and blood collected *via* cardiac puncture, using a sterile syringe and into a heparinized tube. Parasitemia was then calculated by counting the number of parasitized red blood cells (rbc) against total red blood cells. The blood was diluted using 0.9% saline to produce 1 ml blood containing 1 × 10^7^ *P. berghei* infected rbc. 200 µl of the infected blood was then administered intraperitonially to each of test mice. Of the total of 45 female ICR mice, grouped into nine (*n* = 5), 40 were inoculated with the parasites and monitored for parasitemia 72 h post inoculation. Groups 1 to 3 orally received 25 mg/kg of each of the test fractions while groups 4–6 received orally 250 mg/kg of the respective test fraction daily for 4 days. Group 7 received 5 mg/kg artemether-lumefantrine as the positive control for the same number of days. Group 8 which served as negative control A, was inoculated with 1 × 10^7^ *P. berghei* and 10 ml/kg DMSO, while group 9 which served as negative control B, was left un-infected and untreated. Parasitemia and mean survival rate was continuously monitored for 10 days post-infection. Percentage parasitemia was calculated by counting the number of parasitized red blood cells out of five randomly selected fields of view under a light microscope (Leica ICC50 HD microscope, Hamburg Germany) at a magnification of x100. Using the formula:

$$\%\;parasitemia=\frac{number\;of\;parasitized\;RBCs\;}{\left(total\;number\;of\;RBCs\;counted\right)\;}\;X\;100$$

$$\%\;Chemo-suppression=\frac{\left(A-B\right)}{A}X100$$

Where A is the average percentage parasitemia in the negative control group and B is the average percentage parasitemia in the test group.

Mean Survival Time (MST). During the follow-up period, daily mortality and the number of days from parasite inoculation till death were recorded for each mouse in all groups. MST for the various groups was calculated using the formula:

$$MST=\frac{Sum\;of\;survival\;time\;of\;all\;mice\;in\;a\;group\;\left(days\right).}{\;sum\;of\;mice\;in\;a\;group}$$

### 3.3 Thin Layer Chromatography of crude extracts and fractions

Thin Layer Chromatography was run on a 0.25 mm Aluminium oxide DC—Fertigplatten ALOX-25 (Macherey-Nagel, Germany) glass TLC plate and viewed under UV light of 254 nm. Prior to TLC analysis, silica gel layers were pre-treated with 0.02 M sodium acetate. Then the plates were dried out at 50°C in an oven for 5 min. Stock solutions of test samples were applied at the start of the plates manually by means of a micro syringe in volume of 0.2 μl. The layers with applied analytes were developed with petroleum ether-chloroform-methanol [PE: CHCl3: MeOH (1:4:0.5)] as mobile phase at laboratory temperature. Staining was done with iodine vapour from an iodine vapour tank.

### 3.4 Statistical analysis

GraphPad v8.0 and Microsoft Excel 2019 were used for statistical analysis. The mean variations were compared using the Mann-Whitney t-test, one-way ANOVA, or Kruskal-Walli’s test, depending on the normality of the data. Dunnett’s test was performed as a post-hoc at a 95% confidence range. Results were expressed as mean ± S.D. *p* < 0.05 was considered statistically significant. GraphPad was used to create all the graphs.

## 4 Results

### 4.1 Acute toxicity study

Acute toxicity studies of orally administered fractions, ACL1:1, ACL100%, and CPL100% in ICR mice indicated an LD_50_ score above 2000 mg/kg. Also, oral administration of all three fractions did not result in any clinical signs of toxidromes such as weight loss, abnormality in movements, salivation, difficulty in breathing, frequency and consistency of stool and mortality as compared to the control group. Post-mortem examination of the viscera, kidney, liver, brain, spleen, and heart revealed no visible abnormal changes in the treatment groups in comparison with the control. Organ weights did not show significant differences between the test groups and control during the acute studies. Haematology and serum biochemistry analyses at the end of the 14-day study did not reveal statistically significant differences, albeit slight variations in values between the test and control groups as shown in Table 1, 2 *.*

**TABLE 1:** Effect of fractions ACL1:1, CPL100% and ACL100% on hematological parameters in the acute toxicity study.

Parameter (Unit)	Control	ACL1:1	CPL100%	ACL100%
WBC (×10^3^u/L)	5.76 ± 1.92	5.03 ± 1.42	4.55 ± 1.86	7.675 ± 1.31
RBC (×10^6^ul)	7.17 ± 0.75	8.08 ± 0.08	7.64 ± 1.25	7.85 ± 0.21
HGB (g/dl)	11.64 ± 1.20	13.70 ± 0.26	12.07 ± 1.20	12.30 ± 0.28
HCT (%)	0.39 ± 0.03	8.08 ± 0.08	0.39 ± 0.07	21.06 ± 29.20
MCV (fL)	53.74 ± 1.92	52.50 ± 1.00	51.96 ± 1.33	52.40 ± 2.12
PLT (×10^3^ul)	458.80 ± 377.50	1087 ± 367.00	447.90 ± 399.1	1234 ± 203.60
LYM%	70.46 ± 13.47	83.23 ± 8.32	63.47 ± 9.60	72.95 ± 2.05

ACL, 1:1, ACL, 100% and CPL, 100% are the chloroform fractions of the hydro-ethanol extracts of *Alchornea cordifolia* leaves, absolute ethanol extracts of *Alchornea cordifolia* leaves and absolute ethanol extracts of *Carapa procera* leaves, respectively, *Data expressed as Mean ± S. D (n= 3). Mann-Whitney t-test, significance set at p < 0.05. WBC, White blood cell; RBC, Red blood cell; HGB, Haemoglobin; HCT = Hematocrit; MCV, Mean Corpuscular volume; PLT, Platelet; LYM, lymphocyte*.

**TABLE 2:** Effect of fractions ACL1:1, CPL100% and ACL100% on biochemical parameters in the acute toxicity study.

Parameters	Control	ACL1:1	CPL100%	ACL100%
Bilirubin Total (μmol/L)	8 ± 5.29	6.00 ± 2.00	2.32 ± 0.46	9 ± 1.41
Direct Bilirubin (μmol/L)	2.67 ± 0.58	2.00 ± 1.00	1.07 ± 0.24	2.00 ± 0.00
Alkaline Phosphatase (IU/L)	176.70 ± 29.00	158.00 ± 1.00	332.30 ± 75.50	206.00 ± 72.10
ALT (IU/L)	215.30 ± 158.90	178.00 ± 1.00	87.77 ± 50.90	45.50 ± 23.30
AST (IU/L)	599.00 ± 407.20	672.00 ± 1.00	182.20 ± 95.50	181.00 ± 114.60
GGT (IU/L)	21.00 ± 1.00	21.00 ± 0.00	15.00 ± 0.00	14 ± 0.00
Total Protein (g/L)	55.33 ± 3.79	58.67 ± 1.53	64.54 ± 5.85	59.00 ± 5.00
Creatinine (IU/L)	110.00 ± 1.00	46.00 ± 21.66	40.46 ± 3.65	27.00 ± 0.00
Urea	8.00 ± 1.00	6.40 ± 1.30	6.04 ± 1.02	6.20 ± 0.85
μmol/L				

ACL, 1:1, ACL, 100% and CPL, 100% are the chloroform fractions of the hydro-ethanol extracts of *Alchornea cordifolia* leaves, absolute ethanol extracts of *Alchornea cordifolia* leaves and absolute ethanol extracts of *Carapa procera* leaves, respectively. *Data expressed as Mean ± S. D (n= 5). Mann-Whitney t-test, significance set at p < 0.05. ALT, Alanine aminotransferase. ALT: Alanine transaminase. AST, Aspartate aminotransferase; GGT, Gamma-glutamyl transferase*.

### 4.2 SUB-ACUTE toxicity studies

**FIGURE 1:**
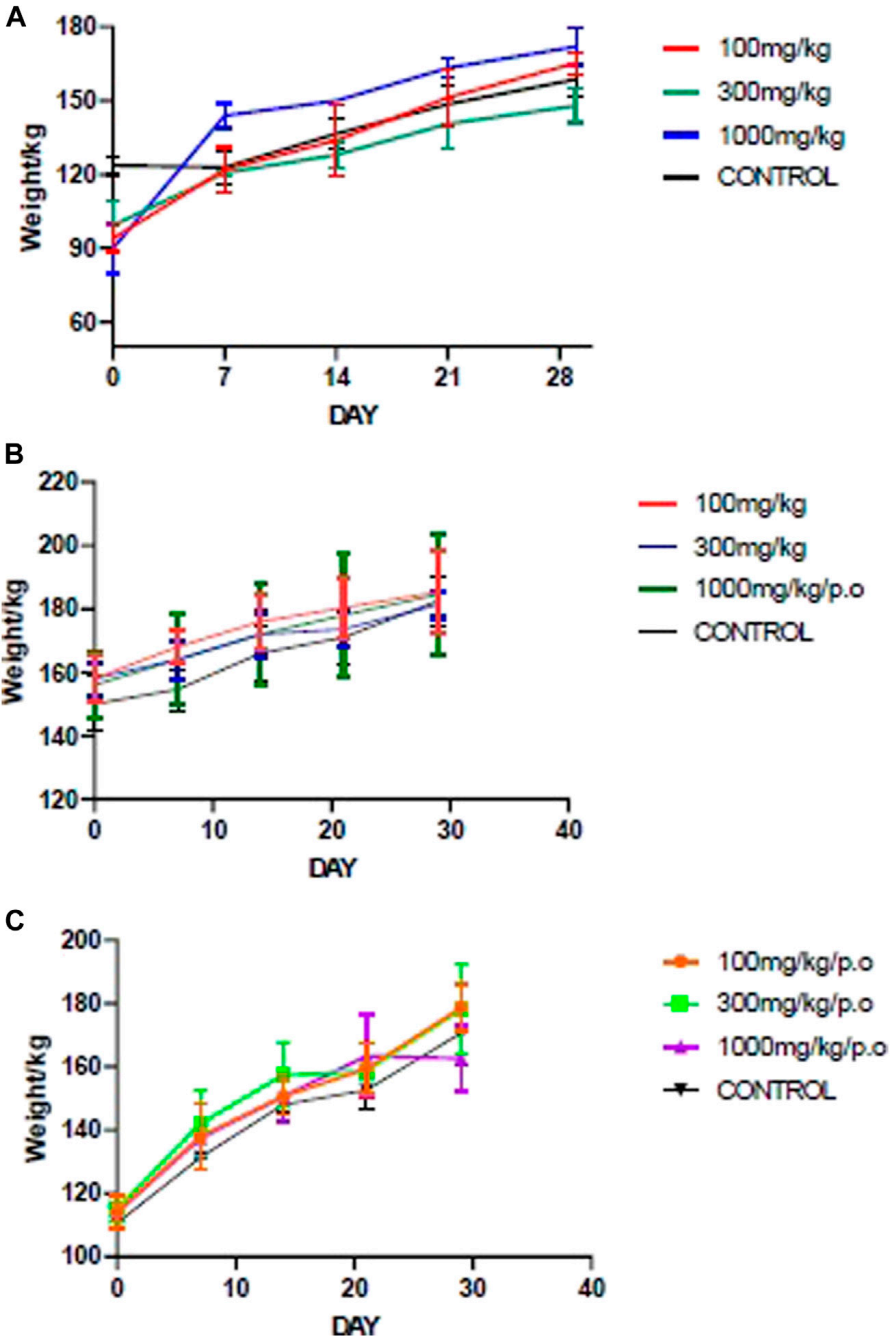
Mean body weight changes during the sub-acute studies of the fractions. **(A)** ACL1:1, **(B)** ACL100%, **(C)** CPL100%. ACL 1:1, ACL 100% and CPL 100% are the chloroform fractions of the hydro-ethanol extracts of *Alchornea cordifolia* leaves, absolute ethanol extracts of *Alchornea cordifolia* leaves and absolute ethanol extracts of *Carapa procera* leaves, respectively.

All animals showed healthy weight gain throughout the 28 days of repeated dose administration. There was a general upward weekly weight gain at the three doses and in all the fractions (Figure 1) Furthermore, there was no dose-related effect found throughout physical and behavioral examination of the individual animals, Haematology, and serum biochemistry analysis at the end of the 28-day study did not reveal any statistically significant differences at *p* < 0.05.

### 4.3 Histopathology

**FIGURE 2:**
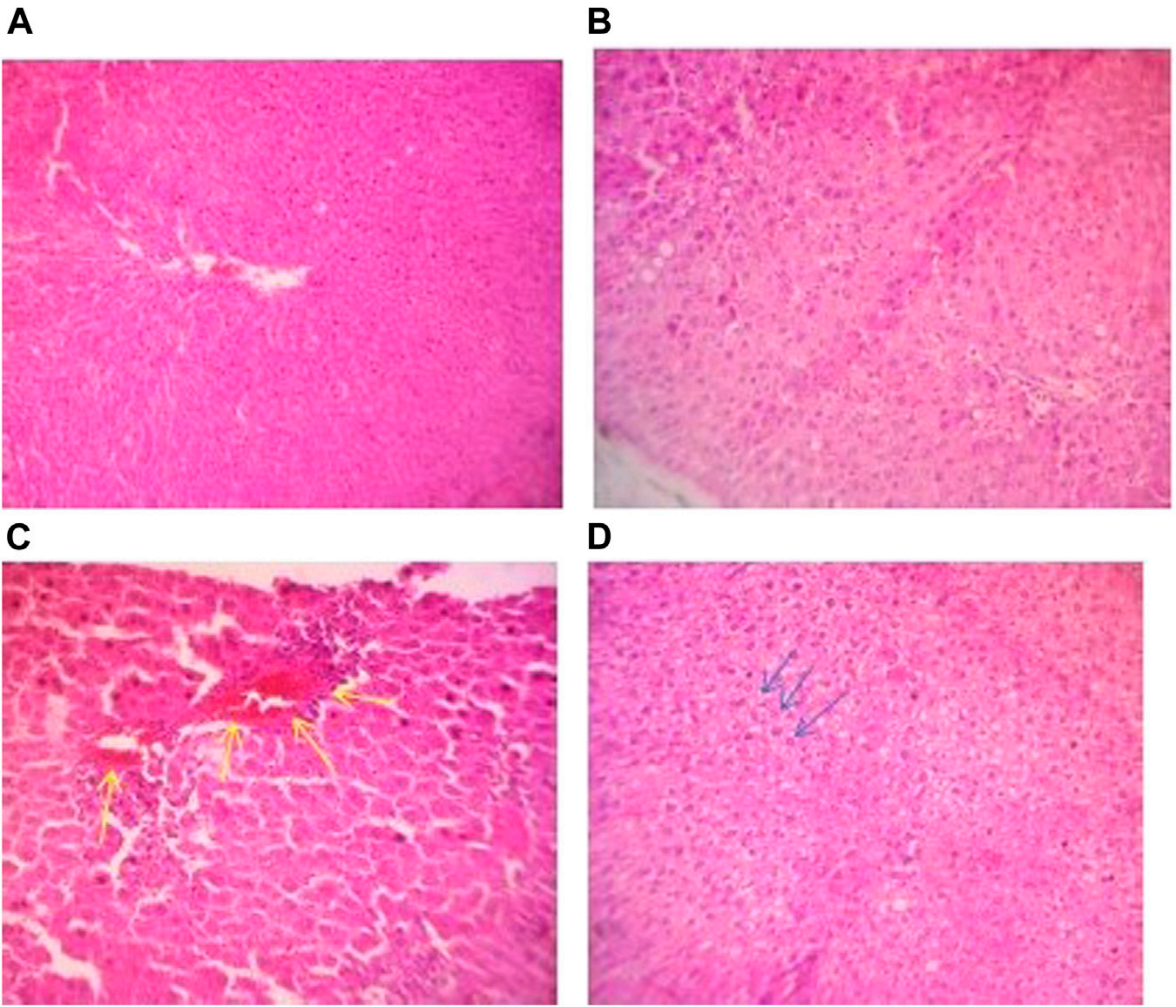
**(A)** Photomicrograph of liver section of a control rat in subacute toxicity study exhibiting normal hepatocytes and sinusoids (H&E stain, X100). **(B)** Photomicrograph of liver section of a rat in subacute toxicity of CPL 100% at 100 mg/kg showing no notable abnormality (H&E stain, X100). **(C)** Photomicrograph of liver section of a rat in subacute toxicity study of CPL 100% at 1000 mg/kg showing piecemeal necrosis showed in yellow arrow (H&E stain, X100). **(D)** Photomicrograph of liver section of a rat in subacute toxicity study of ACL 100% undergoing hydropic change shown with blue arrows.

The livers and kidneys of the control groups and the ACL1:1 fraction showed no signs of necrosis or inflammation. CPL100% at 100 mg/kg and 300 mg/kg showed no signs of toxicity, but the highest dosage, 1000 mg/kg resulted in piecemeal necrosis as indicated in Figure 2B. Similarly, the highest dosage 1000 mg/kg for CPL100% and ACL100%, resulted in a hydropic change in the liver as shown in Figure 2C (yellow arrows) and 2D (blue arrows).

### 4.4 Antiplasmodial assessment

**FIGURE 3:**
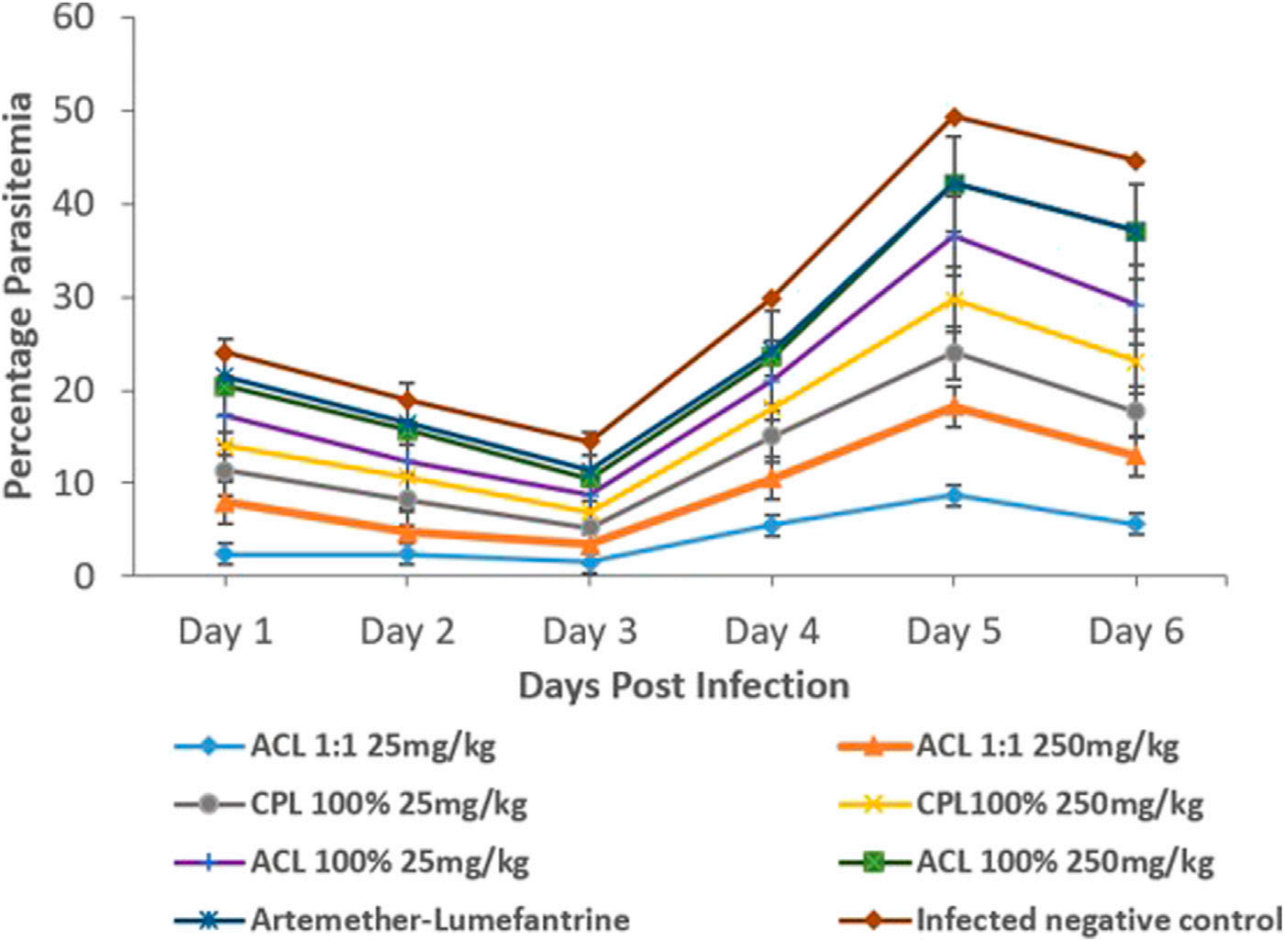
Parasitaemia levels between treatment groups and controls (One-way ANOVA, followed by Dunnet’s post hoc test). ACL 1:1, ACL 100% and CPL 100% are the chloroform fractions of the hydro-ethanol extracts of *Alchornea cordifolia* leaves, absolute ethanol extracts of *Alchornea cordifolia* leaves and absolute ethanol extracts of *Carapa procera* leaves, respectively.

Assessment of curative abilities of the fractions against a positive control (artemether-lumefantrine) lasted for 6 days. After the fourth day of treatment, no parasites were recorded for the positive control group. All other treatment groups showed a steep increase in parasitemia throughout the treatment phase Illustrated in Figure 3; Table 3 *.* All animals of the positive control and CPL 100% 250 mg/kg group survived the 11-day study. However, at least one animal died from all other treatment groups by the end of the study, with ACL 1:1 25 mg/kg having the least survival time of 8.6. The CPL 100% 250 mg/kg treatment group showed the highest chemo-suppression rate of 26.27%.

**TABLE 3:** Mean Parasitemia, Chemo-suppression and Mean Survival Time of the chloroform fractions.

Parameter	Artemether-lumefantrine	ACL 1:1 25 kg/mg	ACL 1:1 250 kg/mg	CPL100% 25 kg/mg	CPL100% 250 kg/mg	ACL 100% 25 kg/mg	ACL 100% 250 kg/mg	Negative control
Mean parasitemia	0.8774	4.358	5.276	3.964	3.487	3.753	4.038	4.755
% Chemosuppression	81.55	8.35	-11	16	26.27	21.1	15.1	
Mean survival time	11	8.6	10.4	10.8	11	10.4	10.4	9.6

ACL, 1:1, ACL, 100% and CPL, 100% are the chloroform fractions of the hydro-ethanol extracts of Alchornea cordifolia leaves, absolute ethanol extracts of *Alchornea cordifolia* leaves and absolute ethanol extracts of *Carapa procera* leaves, respectively.

### 4.5 Similarity assessment of decoctions and chloroform fractions

**FIGURE 4:**
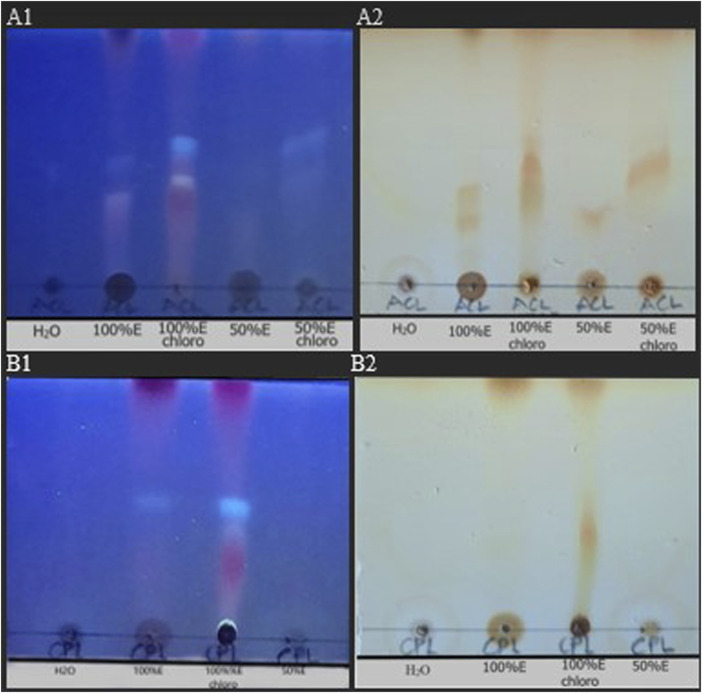
TLC chromatogram of crude extracts and fractions of Alchornea cordifolia leaves (ACL). and *Carapa procera* leaves (CPL). **(A)** ACL aqueous extract (ACL H_2_0), ACL 100% ethanol extract (ACL100%E), ACL 100%E Chloroform fraction (ACL100%E chloro), ACL 50%Etoh:50% aqueous extract (ACL 50%E), ACL 50%E Chloroform fraction (ACL 50%E chloro). **(B)** CPL aqueous extract (CPL H20), CPL 100% ethanol extract (CPL 100%E), CPL 100%E Chloroform fraction (CPL100%E chloro), CPL 50% ethano1:50% aqueous extract (CPL 50%E). Stationary phase: glass-backed precoated silica gel plates (Merck) impregnated by 0.02 M sodium acetate; Mobile phase: PE: CHCI3: MeOH (1:4:0.5) under UV light of 254 nm **(A1,B1)** and stained with iodine vapour **(A2,B2)**.

Decoctions of the leaves of the two plants, as used in traditional settings, was compared to the chloroform fractions assessed in this study. The chromatogram shows more components in the chloroform fractions than the respective crude extracts (Figure 4).

## 5 Discussion

Malaria remains a devastating disease, exerting a high burden on sub-Saharan Africa and children under 5 years of age being the most affected population (World Health Organization, 2018). Over centuries, science has validated the use of plants as sources for medicine through empirical means. Plants, by virtue of their wide array of chemical compounds are potentially toxic (Li et al., 2005). Some plants that have been widely used for therapeutic purposes have been shown to cause damage with chronic use (Ernst, 2003a). This study therefore sought to, as part part of investigating various fractions of varying polarity to enable the determination of the most active, assessed the toxicological and antimalarial effects of the chloroform fractions of *Carapa procera* and *Alchornea cordifolia* in murine animal models*.* We have also provided in this study the thin layer chromatogram of the test fractions and the traditionally used form of the plant ectracts.

The acute and sub-acute toxicity studies indicated that *A. cordifolia* and *C. procera* are relatively safe. In the acute toxicity study, the highest dose of 2000 mg/kg administered orally recorded no death in the animals. LD_50_ score was therefore set at above 2000 mg/kg. This is because orally administered products that do not cause toxicity at 2000 mg/kg dosage or higher are considered highly safe in animals (Organisation for Economic Co-operation and Development, 2002). During the sub-acute toxicity study, absolute body weight gain for both control and treated groups was normal and followed an upward trend during the 28-day sub-acute toxicity studies (Figure 1).

Alteration in hematopoietic and/or biochemical parameters of the body serves as targets of toxic compounds and can therefore be indicative of toxicity before clinical studies in human subjects (Naghibi et al., 2014). Analysis of hematopoietic parameters, including white blood cells (WBCs), red blood cells (RBCs), Hemoglobin (HGB), lymphocytes and platelets did not reveal any significant reductions. The relatively similar levels of RBCs and HGB levels could indicate that intake of the fractions did not cause anemia or hematological disorders. Biochemical parameters were assessed to detect nephrotoxicity and hepatotoxicity since liver and kidney dysfunctions have been associated with the use of medicinal plants (Adewunmi et al., 2001). Aspartate transaminase (AST) and alanine transaminase (ALT) are markers of liver function. High AST, ALP and ALT levels suggest hepatotoxicity. From Figure 1, 2, it can be observed that there were no significant differences between the test and control groups of ACL1:1, ACL100% and CPL100% chloroform fractions. Assessment of creatine and urea levels, major indicators of renal function were not affected in any of the treatment groups.

*A. cordifolia* toxicity study results obtained in this study are in line with that of two published studies (Ajibade and Olayemi, 2015; Ezeokeke et al., 2017), and the toxicity study findings of C. *procera* aligns with a previously published study (Koama et al., 2021). In all three studies, extracts of both plants were found to show normal levels of hematological and biochemical parameters, compared to controls.

Histopathological findings did not correlate with biochemistry findings of the highest doses, 1000 mg/kg in the cases of CPL100% and ACL100% fractions. At this high dose, both extracts showed early signs of liver degeneration. This is in concordance with a study where administration of *A. cordifolia* extract at 750 mg/kg caused the liver injury (Banzouzi et al., 2002). These signs of degeneration may have occurred too early to be detected in liver function tests and therefore may be reversible when the administration is stopped. Herbal medicines are known to contain numerous active compounds, responsible for different pharmacological functions. It is therefore possible that toxicants present in the two fractions were concentrated at the high dosage of 1000 mg/kg, resulting in the observed liver degeneration. Kidney sections of the test and control groups did not indicate any nephrotoxicity, as observed in the kidney function tests. The flavonoid content in both *A. cordifolia* and *C. procera* may be responsible for the protection against kidney damage (Boakye-Yiadom et al., 2021; Owusu et al., 2021). Structurally, the bowman’s capsule remained intact in the control and all test groups.

Stem, bark, and leaf decoctions of plants of the family *Meliaceae* have been widely used as antimalarials in west Africa, including *Azadiracta indica*, commonly called neem, *Khaya senegalensis* and *Khaya grandifoliola* (Obih and Makinde, 1985; Adebayo et al., 2003; Bumah et al., 2005). In the present study, ICR mice were used to assess the anti-plasmodial activity of *Alchornea cordifolia* and *Carapa procera* fractions. The CPL 100% chloroform fraction displayed a moderate dose-dependent increase in anti-plasmodial activity according to the anti-plasmodial activity scale (Muñoz et al., 2000), with 16% for the 25 mg/kg dose and a corresponding increase in chemo-suppression to 26.27% when the dose was increased to 250 mg/kg. A study by Koama et al. (Ezeokeke et al., 2017) found a 23.1% reduction when 250 mg/kg of macerated ethanol extract of *C. procera* was orally administered. The group also employed Soxhlet ethanol extraction of *C. procera* and found that it exhibited an appreciably higher anti-plasmodial activity of 46.3% and 56.7% at 100 and 250 mg/kg respectively. The same study found, through phytochemical screening, the presence of flavonoids in *C. procera*. Flavonoids have been found to show anti-malarial activity (Imam et al., 2013). The increase in chemo-suppression by the chloroform fraction relative to the macerated ethanol extract observed in this study may be because of solvent polarity influencing extraction yield (Liu et al., 2009), and thus, flavonoids can be better extracted in chloroform and will therefore, have a higher yield to drive anti-plasmodial activity. The method of extraction could be responsible for the higher chemo-suppression in the above study where Soxhlet extraction was applied, compared to the maceration method of extraction used in this study. All animals from the CPL 250 mg/kg group survived the duration of the experiment and one from the CPL 25 mg/kg group died.

In the present study, the ACL1:1 chloroform fraction resulted in a low MST of 8.6 and showed weak anti-plasmodial activity of 8.3% at 25 mg/kg and an even weaker reduction to −11% when the dosage was increased to 250 mg/kg. This reduction may be explained by the saturation of plasma protein binding sites (Bohnert and Gan, 2013). The ACL 100% fraction showed moderate anti-plasmodial activity at 25 mg/kg of 21.1% which reduced to 15.1% upon increasing the dosage to 250 mg/kg. Both dosages also caused the same MST of 10.4. The variation in anti-plasmodial activity of the ACL 1:1 and ACL 100% fractions may be due to the extraction process. The former was done with a 50:50 volume of ethanol and distilled water before fractionation, whereas the latter was done with 100% ethanol. Since the extracting solvent concentration affects the yield of active compounds, it is possible the 100% ethanol extraction yielded more of the active compounds postulated to be responsible for antimalarial activity (Obih and Makinde, 1985). The TLC chromatogram gives further credence to this following the presence of more components in the fractions than the respective crude extracts.

Results from this study have shown that chloroform fractions of the leaf extracts of *Alchornea cordifolia* and *Carapa procera* are relatively non-toxic to hematological and renal systems. At high doses, however, *Carapa procera* is potentially hepatotoxic with long-term use. Interestingly, both plants showed moderately significant *in-vivo* activity against *P. berghei* with animals demonstrating a higher survival rate in the *C. procera* group than the *A. cordifolia* and therefore lends possible efficacy to the widespread use of both plants as antimalarials. This study will form the basis for further development of these indigenous plant medicines into efficacious anti-malarial.

## Data Availability

The original contributions presented in the study are included in the article/supplementary material, further inquiries can be directed to the corresponding author.
